# The Pathogenesis and Treatment of Hidradenitis Suppurativa

**DOI:** 10.7759/cureus.49390

**Published:** 2023-11-25

**Authors:** Erica R Agnese, Nicole Tariche, Amit Sharma, Raj Gulati

**Affiliations:** 1 Dermatology, Lake Erie College of Osteopathic Medicine, Elmira, USA; 2 Obstetrics and Gynecology, Lake Erie College of Osteopathic Medicine, Elmira, USA; 3 General Surgery, Lake Erie College of Osteopathic Medicine, Elmira, USA

**Keywords:** multimodal intervention, hidradenitis suppurativa risk factors, hidradenitis suppurativa, hidradenitis suppurativa treatment, hidradenitis suppurativa pathogenesis

## Abstract

Hidradenitis suppurativa (HS) is a multifactorial disease involving the skin and subcutaneous tissues characterized by deep-seated, painful nodules and abscesses with draining sinus tracts. It affects mostly younger individuals between the ages of 18 and 34. The discomfort and embarrassment that patients affected by HS experience negatively impact their daily lives. It is associated with decreased quality of life and high rates of comorbid depression and anxiety. The rate of depression in HS was reported to be as high as 26%. Its pathogenesis is multifactorial and as such requires a multimodal approach to treatment, which subsequently is reviewed here. Moreover, the pathogenesis of HS is complex and only partially understood. Autoinflammation is the key driver of disease development and is linked with dysregulated inflammasome activation with the subsequent production of inflammatory cytokines. Genetics and cutaneous microbiome play a role in the development of chronic inflammation and lesion formation. Risk factors such as obesity, metabolic syndrome, diabetes, and smoking also add to the systemic inflammation. Targeting these risk factors is a key aspect of the treatment of HS. Lifestyle modifications are used in conjunction with pharmacotherapy and procedures to effectively manage the disease.

## Introduction and background

Introduction

Hidradenitis suppurativa (HS) is a multifactorial disease involving the skin and subcutaneous tissues that is characterized by deep-seated, painful nodules and abscesses with draining sinus tracts. It affects mostly younger individuals between the ages of 18 and 34 [1]. The discomfort and embarrassment patients affected with HS experience are disruptive to their lives and are associated with low quality of life and high levels of comorbid depression and anxiety [2,3]. A study assessing the quality of life of HS patients using the Dermatology Life Quality Index (DLQI) showed disease severity was correlated with higher DLQI scores and greater impairment [3]. The most commonly affected areas are intertriginous regions including the axilla, inframammary folds, inguinal region, and gluteal clefts [2,4,5].

The pathogenesis of HS is still being elucidated, but recent research, which includes studies analyzing the molecular and biochemical basis as well as the microbiome in HS, has made great progress [2,4]. Further identification of the complicated factors that contribute to HS is crucial for developing more effective therapies. The pathogenesis of HS involves not only systemic autoinflammation, which is exacerbated by numerous risk factors, but it is also characterized by cutaneous microbiome dysregulation and genetic contributions. This paper aims to discuss the current understanding of the pathogenesis of HS after an extensive review of the literature.

Methods

A search of the recent literature was conducted using PubMed and Ovid. The terms searched included "Hidradenitis Suppurativa", "Hidradenitis Suppurativa Pathogenesis", and "Hidradenitis Suppurativa Treatment". 

Epidemiology

The prevalence of HS is estimated to be anywhere from 0.00033% to 4.1% [2,4]. It shows a female predominance among Western countries with a ratio of 1.3:1 women to men affected [1,2,4,6], while Eastern countries show a male predominance [5,7]. The highest incidence is found among African American women [1,2,4], and patients of lower socioeconomic status are disproportionately affected by the disease [1,8].

In the United States, the rate of hospitalization of patients with HS increased from 3,145 in 2008 to 4,170 in 2017 [1]. 12.7% of patients hospitalized with HS had major or extreme loss of function [1]. There has been a 46.8% increase in HS hospitalizations; however, this may be attributed to an increase in HS research, greater awareness of the disease, and improved diagnostics, but no studies have been done proving any of these correlations [1].

Patients affected by HS report lower quality of life [2,3,9]: the disease disrupts their activities of daily living by negatively impacting their work and school lives [3]. There are high rates of comorbid anxiety and depression [2,6,9,10] and higher rates of completed suicide among HS patients when compared to a control population [2,10]. Rates of depression among HS patients were reported to be as high as 26% [10]. Depression in HS patients is correlated with disease severity and treatment outcomes [11]. A survey conducted among primary care physicians in the United Kingdom showed suboptimal management of pain and psychological symptoms of HS patients, which reflects a need for further education on HS management to improve patients' quality of life [12].

## Review

Pathogenesis

Previously, the inciting event in the pathogenesis of HS was thought to center around the inflammation of the apocrine glands. However, newer evidence suggests the inciting event to be follicular hyperkeratosis of the pilosebaceous-apocrine gland unit. The sebaceous glands secrete oils that are called sebum, while the apocrine glands drain sweat and oil through a duct that passes through the hair follicle [4]. Follicular hyperkeratosis results in follicular plugging and eventual rupture, releasing follicular contents [2,7]. This activates the innate immune system and in turn leads to secondary inflammation of the apocrine glands [2,6,7,13]. 

The hyperkeratosis seems to be driven by the excessive activation of inflammatory pathways and aberrant cytokine signaling [2,4,6]. Activation of the inflammasome, which is a group of proteins that responds to stimuli, triggers the production and release of pro-inflammatory cytokines. The cytokines, in turn, contribute to both the primary inflammation and the perpetuating inflammation in HS [7,13]. The inflammasome activates a pro-inflammatory cascade, which regulates tissue repair and death [13]. Among other functions, it releases caspase-1, which cleaves IL-1 from its inactive form to its active form of IL-1 beta. The overactivation of macrophages and Th17 cells leads to the increased release of IL-23, tumor necrosis factor (TNF)-alpha, and IL-17 [14]. TNF-alpha and IL-17 levels are directly correlated with disease severity [4].

The cellular infiltrate of HS lesional tissue in moderate to severe disease shows high levels of inflammatory cells especially Th1 and Th17 cells and neutrophils [2,6,7,13]. Studies of the transcriptome analyzing the RNA in HS lesional tissue showed the upregulation of genes associated with these inflammatory cells, including CD-3 and CD-25 (T cells) and neutrophils [7]. 

**Figure 1:**
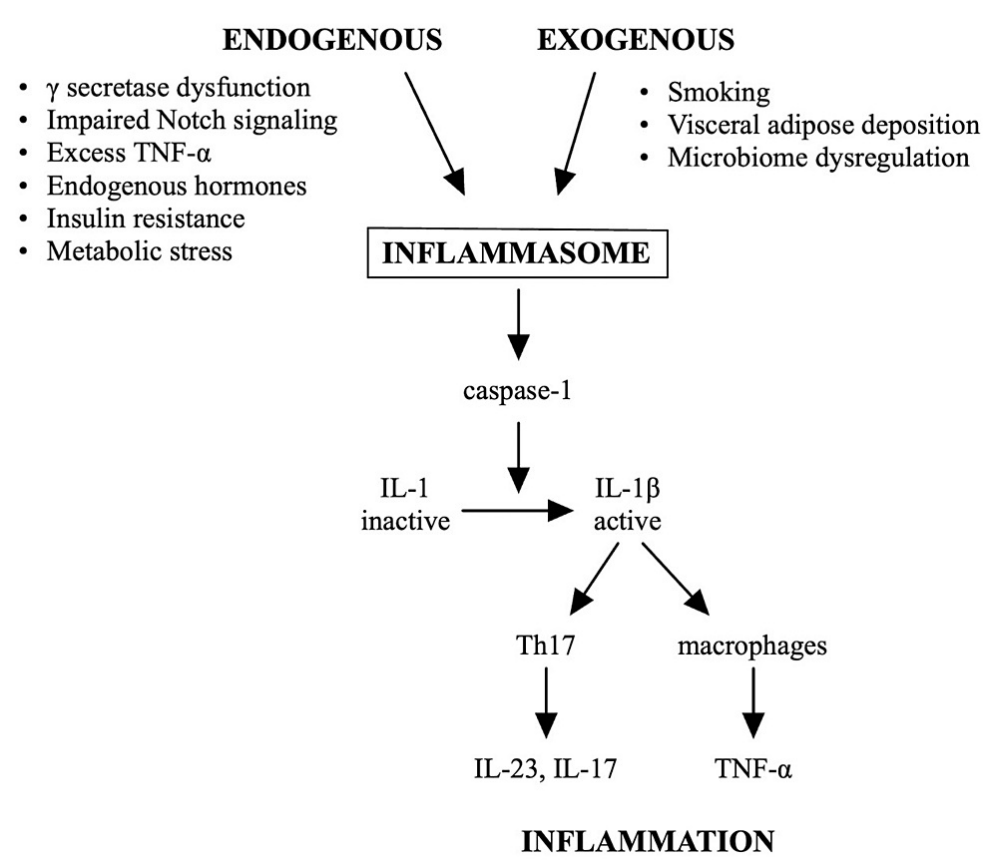
The pathogenesis of hidradenitis suppurativa Image Credit: Nicole Tariche

Inflammasome activation can be in response to endogenous or exogenous stimuli. Endogenous sources include gamma secretase dysfunction, impaired Notch signaling, excess TNF-alpha, endogenous hormones, insulin resistance, and metabolic stress. Exogenous sources include smoking, visceral adipose deposition, and dysregulation of the microbiome [13]. The triggers for inflammasome activation are illustrated in Figure 1 and discussed in further detail throughout the remainder of this article. 

Risk factors

Risk factors for HS contribute to the overall systemic pro-inflammatory state associated with the disease. The most common risk factors include obesity, hyperlipidemia, cardiovascular disease, metabolic syndrome, diabetes mellitus, genetics, and smoking. Hormonal imbalance has also been implicated in HS pathogenesis although the extent of its contribution is still unclear. Risk reduction through weight loss is correlated with a decreased severity of disease [4].

Obesity

Obesity contributes to chronic inflammation through the dysregulation of adipokines. Biopsies of HS lesional tissue found elevated levels of pro-inflammatory adipokines, such as resistin and chemerin, as well as elevated inflammatory cytokines IL-1 beta and TNF-alpha. In contrast, levels of the anti-inflammatory adipokine, adiponectin, were decreased [4,15,16]. Obesity also results in an abundance of skin folds. The friction at these sites causes cellular damage, which activates an inflammatory response and allows a route of entry for bacteria further contributing to lesion formation in HS [17].

Metabolic Syndrome and Diabetes

Insulin resistance is common among HS patients. Insulin resistance may contribute by promoting a low-level pro-inflammatory state [7,16]. Insulin resistance is associated with similar adipokine dysregulation to obesity, tipping the balance of anti-inflammatory adipokines and pro-inflammatory adipokines towards a pro-inflammatory state. This occurs through the increased expression of resistin and leptin, which are pro-inflammatory, and the decreased expression of adiponectin, which is anti-inflammatory [16]. In addition, insulin resistance also leads to an excess insulin state. The excess insulin binds to insulin-like growth factor-1 (IGF-1) receptors. Insulin binding to IGF-1 receptors on keratinocytes stimulates their proliferation, contributing to hyperkeratosis [18]. 

Insulin-resistant patients with HS who were treated with metformin showed a decrease in disease severity and flares [4,7,8]. Metformin itself has an anti-inflammatory effect. A proposed mechanism for the anti-inflammatory actions of metformin includes the inhibition of mTORC1, which downregulates the Th17 response and decreases levels of IL-6 and TNF-alpha [7,8].

Smoking

Smoking is a common risk factor among patients with HS. There are abundant nicotinic receptors expressed in the axillary sweat glands, keratinocytes, and immune cells. Stimulation of the nicotinic receptors in keratinocytes promotes differentiation and growth, which leads to hyperkeratosis and follicular plugging [7]. TNF-alpha and inflammatory cytokines were found to be higher among smokers, and as previously stated, TNF-alpha levels are directly correlated with disease severity [7].

Smoking promotes the growth of *Staphylococcus aureus* leading to its overgrowth, which disrupts the cutaneous microbiome and contributes to local inflammatory responses [7,13]. Nicotine also reduces the levels of anti-microbial peptides, including hbD2, leaving the cutaneous tissue at risk for further infections [7].

Hormonal Imbalance

HS is also associated with hormonal imbalance. Most patients with HS fail to demonstrate systemic hyperandrogenism, so the effects of hormonal influence on HS may be local rather than systemic [4,13]. The relationship has been established through observations that women tend to have disease flares associated with menses as well as comorbid polycystic ovary syndrome (PCOS). Combined oral contraceptives, spironolactone, and other anti-androgenic agents have been shown to decrease HS flares among women further supporting a hormonal contribution to disease development [4,16,19].

Genetics

Familial forms of HS have been observed, behaving as an autosomal dominant disorder. Mutated genes driving the development of HS are multifactorial; however, the most implicated genes are the ones encoding subunits of the gamma secretase protein. Gamma secretase plays a central role in Notch signaling, which acts to prevent keratinocyte differentiation and growth [14]. Lack of a properly functioning gamma secretase gene leads to aberrant keratinocyte differentiation and growth with subsequent hyperkeratosis.

Loss-of-function mutations in NCSTN, PSEN1, and PSENEN, which encode for subunits of gamma secretase, are implicated in disease development [2,14]. Studies of the NCSTN mutation linked it to the decreased expression of a microRNA known as miR-30a-3p. miR-30a-3p downregulates the expression of RAB31, which is linked to aberrant keratinocyte differentiation [14]. Sporadic cases of HS have shown genetic alterations consisting of single nucleotide polymorphisms in the genes encoding TNF and toll-like receptor 4 [20].

Microbiome

Dysbiosis of the cutaneous microbiome is implicated in contributing to the inflammation driving HS. Samples of HS lesional tissue showed increased *Prevotella* and *Porphyromonas* compared to a predominance of *Cutibacterium* in normal tissue [17]. *Prevotella* in particular has been shown to stimulate a Th17 cell response, which is a strong driver in the development of HS [17].

Biofilm formation was also found to be common in HS lesions, but not in samples of non-lesional tissue [21]. Biofilm formation is driven by *Staphylococcus epidermidis*. Sites of biofilm formation were found to have high levels of CD4+ T cells, which have been suggested to stimulate the production of regulatory T cells, thus contributing to skin dysbiosis [17]. The microbiome is thought to contribute to the pathogenesis of HS by inciting an aberrant immune response rather than a response to an infectious process [8].

Diagnosis and staging

The diagnosis of HS is a clinical one, and three criteria must be met for a diagnosis to be made. These include characteristic lesions, a predilection for flexural sites, and lesion recurrence [2,4,13]. The process of diagnosing HS is lengthy for many patients, taking as long as seven to 10 years in some cases [2,8]. Asking patients about boils in characteristically affected areas has a high sensitivity, specificity, and positive predictive value when screening for HS (90%, 97%, and 96%, respectively) [2]. 

**Figure 2:**
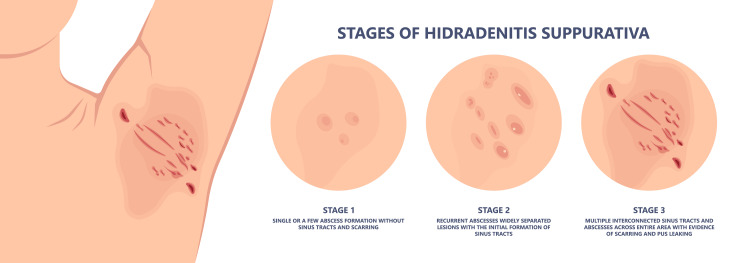
The Hurley stages Image Credit: [24]. Permission was obtained for the use of this image.

**Figure 3:**
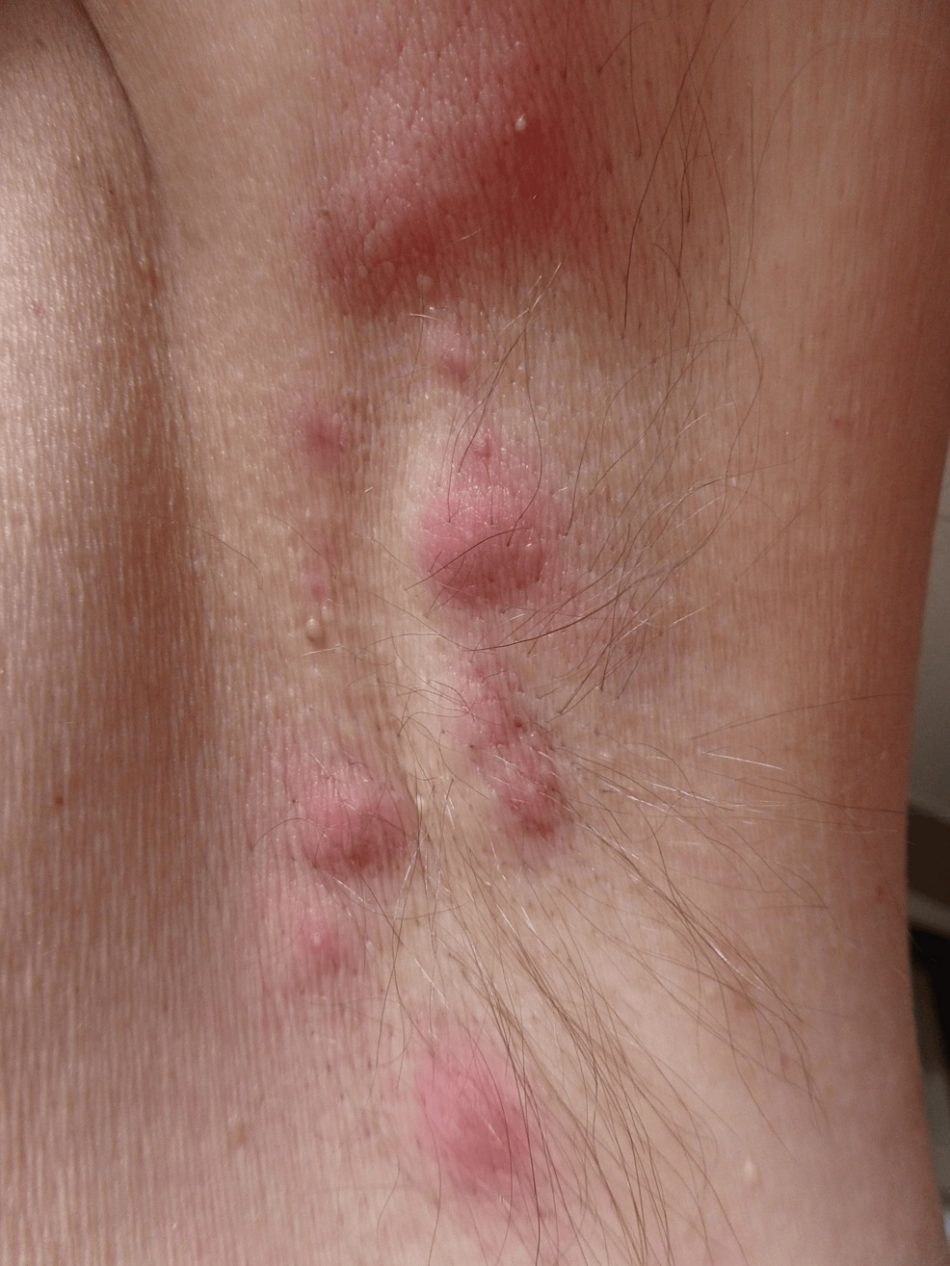
Axillary hidradenitis suppurativa: Hurley stage II Image Credit: [25]. Permission was obtained for the use of this image.

The Hurley staging system, which is illustrated in Figure 2, consists of three levels of disease severity based on clinical findings [2,4,6]. This staging system is used primarily to guide the treatment of the disease and does not accurately assess response to treatment [4,6]. Stage I is characterized by nodules and abscesses without sinus tracts or scarring. Stage II includes one or more sinus tract forming or scarring with the normal skin intervening in addition to nodules and abscesses. An example of Hurley stage II is shown in Figure 3. Stage III is the most severe and includes extensive sinus tracts and scar formation covering the entire affected region, in addition to nodules and abscesses [21,22]. A more dynamic scoring system that is being utilized is the International Hidradenitis Suppurativa Severity Score System (ISH4) [23]. This tool requires counting lesions to assess severity where nodules are assigned 1 point, abscesses 2 points, and sinus tracts 4 points [23]. An ISH4 score of 3 or less is mild disease, 4-10 is moderate disease, and 11 or higher is severe disease [23].

Other more dynamic scoring tools are used to assess treatment response to disease. The modified Hidradenitis Suppurativa Score (mHSS) requires the precise quantification of lesions and consists of four different variables to classify the disease [6]. This allows physicians to follow disease progression and response to treatment [6]. The Sartorius score incorporates the visual pain scale and DLQI, which allows for the monitoring of disease severity and treatment response [5,23]. The Hidradenitis Suppurativa Clinical Response (HiSCR) is commonly used to evaluate response to treatment [23]. It corresponds with a more than 50% reduction in inflammatory lesion count without the formation of new abscesses or sinus tracts compared to the patient's baseline [23].

Treatment

Traditionally, treatment of HS has been guided by the Hurley staging system [4,6,21,22]. For mild HS, Hurley stages I and II, topical therapy is indicated [8,26,27]. Disease management consists of encouraging lifestyle modifications, topical and systemic medications, and procedures [8,21-23].

Managing the Microbiome

Topical therapy is aimed at the cutaneous microbiome, with the first-line therapy for mild localized disease being topical clindamycin [8,21,22]. Intralesional triamcinolone is used for active flares of localized disease [8,22]. For more widespread mild disease, tetracyclines are the first-line therapy [23,27]. However, the benefit of tetracyclines and other antibiotics is thought to be due to an anti-inflammatory effect rather than an anti-bacterial effect [8].

Therapy for Dysregulated Inflammatory Pathways

Hurley stages II and III are treated with more aggressive therapy. This includes immunosuppressants and biologics to target the dysregulated immune response and systemic steroids to target inflammation [8,21,26,27]. Systemic corticosteroids are commonly utilized, but only for acute flares due to the potential of rebound disease with prolonged use [21]. The steroid of choice is prednisolone [21]. The only approved FDA biologic for the treatment of HS was adalimumab (Humira®, AbbVie, North Chicago, Illinois, United States), which is a TNF-alpha inhibitor [8,21,26]. However, secukinumab (Cosentyx®, Novartis, Basel, Switzerland), an IL-17 inhibitor, received FDA approval for the management of moderate to severe HS in October 2023 [28]. Other biologics are being investigated to target the other inflammatory pathways that are implicated in HS [8,21,26]. These biologics being explored are IL-23, IL-1, and JAK inhibitors [8,26]. 

Oral Retinoids

The oral retinoids used in the management of HS are acitretin and isotretinoin [8,21-23,26,27]. The literature is divided on which drug is superior for the treatment of HS, with some studies reporting greater efficacy with acitretin [21,23] and others reporting isotretinoin showed greater benefit [8,22]. Acitretin acts on keratinocytes and decreases hyperkeratosis and has also been demonstrated to decrease the levels of Th-17 cells [8,29]. Isotretinoin is a vitamin A analogue that acts primarily at the sebaceous glands to modulate their secretions [29].

Hormonal Management

While systemic hyperandrogenism is not a common feature of the disease, women with HS, especially those with comorbid PCOS, benefit from hormonal therapy [16,24]. Oral contraceptive pills and anti-androgenic therapies, such as spironolactone, have shown to be of benefit [16,22,23]. Metformin has demonstrated benefits in glycemic control, as well as having anti-inflammatory and anti-androgenic activity [16,22,23].

Procedural Management

Procedures are also employed to target primary lesions [22,23]. Incision and drainage provide rapid relief, but do not resolve the primary lesion [20,26]. It is used for flares of mild to moderate HS [22,30]. Deroofing is used for Hurley stages I and II with lower rates of recurrence when compared with incision and drainage [22,23]. Hurley stages II-III can be treated with wide local excision [22,23]. This treatment is reserved as a last resort for disease that is refractory to pharmacologic treatments and with irreversible tissue damage such as scarring and sinus tract formation [23]. There are variable rates of recurrence with surgical excision, but closure technique can minimize recurrence [22,23,30,31]. 

Multimodality treatment, specifically combining biologic therapy with other modalities of treatment, is proving to be promising and effective in the management of HS. A study evaluating the effects of adalimumab in combination with surgery showed statistically significant results in the improvement of disease severity as well as quality-of-life score in the cohort that received dual therapy when compared to adalimumab alone [32].

Lifestyle Modifications

In addition to treatment with pharmacologic therapy, all patients should be encouraged to make lifestyle changes to minimize disease and comorbidities associated with HS [23,26,27]. Weight loss, smoking cessation, wearing loose-fitting clothing to avoid mechanical irritation, and avoidance of shaving over the affected areas can help to minimize the severity of HS [21,24,26,27]. Due to the complicated nature of the disease and the treatment, it is vital to engage in thorough patient education to improve compliance with treatment regimens [21].

## Conclusions

The pathogenesis of HS is complex and still only partially understood. Autoinflammation is the key driver to disease development and is linked with dysregulated inflammasome activation with the subsequent production of inflammatory cytokines. Genetics and cutaneous microbiome play a role in the development of chronic inflammation and lesion formation. Risk factors such as obesity, metabolic syndrome, diabetes, and smoking also add to systemic inflammation. Strides have been made to further understand how risk factors contribute to the overall inflammation that underpins HS. These strides strongly suggest that lifestyle modifications are an important target in therapy to reduce HS severity. Lifestyle modification, in combination with multimodality treatment, may be the best course of management for the treatment of this complex disease.
